# Relationship between fatty pancreas and hypertriglyceridemic waist phenotype: a cross-sectional study

**DOI:** 10.1038/s41598-020-78883-1

**Published:** 2020-12-14

**Authors:** Xiaoping Yu, Dan Wang, Weiming Xiao, Xinlin Shi, Qiang She, Hongguang Sun, Tingyue Qi, Renyan Xu, Guiqing Li, Xinnong Liu, Weijuan Gong, Zhigang Yan, Yanbing Ding, Guotao Lu

**Affiliations:** 1 Institute of Digestive Diseases, The Affiliated Hospital of Yangzhou University, Yangzhou University, No. 386 Hanjiang Median Road, Yangzhou, 225000 Jiangsu People’s Republic of China; 2 Department of Health Promotion Center, The Affiliated Hospital of Yangzhou University, Yangzhou University, Yangzhou, Jiangsu People’s Republic of China; 3 Department of Ultrasound, The Affiliated Hospital of Yangzhou University, Yangzhou University, Yangzhou, Jiangsu People’s Republic of China; 4 Pancreatic Center, Department of Gastroenterology, The Affiliated Hospital of Yangzhou University, Yangzhou University, Yangzhou, Jiangsu People’s Republic of China; 5 Jiangsu Co-Innovation Center for Prevention and Control of Important Animal Infectious Diseases and Zoonoses, College of Veterinary Medicine, Yangzhou, 225001 People’s Republic of China

**Keywords:** Metabolic disorders, Gastroenterology

## Abstract

Hypertriglyceridemic waist phenotype (HTWP) and its quantitative indicator, waist circumference-triglyceride index (WTI), are common quantitative indices of visceral obesity and are closely related to metabolic diseases. The purpose of this study was to investigate the relationship between fatty pancreas (FP) and HTWP in China. FP was diagnosed using trans-abdominal ultrasonography in all participants. According to the waist circumference and serum triglyceride levels, the participants were divided into four phenotype groups: normal waist circumference-normal triglyceride, normal waist circumference-elevated triglyceride, elevated waist circumference-normal triglyceride, and elevated waist circumference-elevated triglyceride (indicating HTWP). Clinical characteristics and biochemical indices were compared among the groups. Receiver operating characteristic (ROC) curves were used to evaluate the utility of WTI as a reference factor for FP screening. The HTWP group had a higher prevalence of metabolic syndrome (84.2%), FP (10.4%), fatty liver (64.5%), and hypertension (15.8%) than the other three phenotype groups. The occurrence rate of HTWP and the median WTI were significantly higher in participants with FP than in those without FP (54.7% vs 21.0%, 222 ± 135 vs 142 ± 141, *p* < 0.001). In the ROC curve analysis, when the maximum area under the curve was 0.746, the WTI was 107.09 and the corresponding sensitivity and specificity were 90.6% and 51.9%, respectively. HTWP is closely associated with FP and can be used as a reference factor for FP screening.

## Introduction

Obesity easily leads to the accumulation of adipose tissue in the body^1,2^. Central obesity, characterised by a large waist circumference (WC), is an especially obvious condition. The accumulation of adipose tissue in specific organs and tissues, such as the heart, liver, and pancreas, is referred to as “ectopic fat deposition”^3^. Fatty pancreas (FP) is an excessive deposition of fat in pancreatic tissue, also known as pancreatic steatosis, pancreatic fatty infiltration, or non-alcoholic FP disease^4^.

FP has no specific clinical manifestations and is diagnosed in most individuals on physical examination. Ultrasound and imaging studies have become the primary methods for diagnosing FP, including ultrasonography, computed tomography (CT), and magnetic resonance imaging (MRI). MRI can sensitively evaluate tissue fat content and is a good standard method for detecting pancreatic steatosis^5^. However, because of health economics and other factors, some new methods for the early diagnosis of FP are still warranted.

The occurrence of FP has been reported to be associated with older age, obesity, hyperlipidaemia, fatty liver (FL)^6^, and type 2 diabetes (T2DM). However, these findings remain controversial^7–13^. At present, the clinical risk factors for FP are unclear.

Lemieux et al. first proposed the concept of the hypertriglyceridemic waist phenotype (HTWP) in 2000, which was defined as elevated serum triglyceride (TG) level and elevated WC^14^. In practice, WC is an easily reproducible anthropometric measure, and WC measurement is the simplest method for assessing abdominal fat accumulation and the associated cardiovascular risk. In addition, WC has been widely used in recent years as a simple, low-cost index of visceral adipose tissue deposition in the abdomen^15^. However, WC alone cannot distinguish the extent of visceral adipose tissue and subcutaneous adipose tissue accumulation in the abdominal cavity^16,17^. Therefore, researchers have suggested that WC and TG level should be included as clinical indicators of visceral fat accumulation^17^. Substantial studies have shown that HTWP may also be used as an indicator for the early diagnosis of coronary heart disease, hypertension^18–20^, metabolic syndrome (MetS)^21^, and T2DM and its complications^19,22–25^ in adults^26–30^ and children^21^.

At present, no study has used HTWP to screen for FP. Therefore, our study aimed to prospectively investigate the relationship between HTWP and FP.

## Methods

### Ethics, consent, and permissions

This study conformed to the ethical principles of the Declaration of Helsinki. Informed consent was obtained from all participants when they underwent waist measurements, and this study was approved by the Ethics Committee of the Affiliated Hospital of Yangzhou University.

### Subjects recruitments

This was a cross-sectional study. The study population, consisting of medical staff (including current and former employees) of the Affiliated Hospital of Yangzhou University, was selected in 2016, as described previously^6^. Briefly, the exclusion criteria were as follows: acute or chronic inflammatory disease; previous diagnosis of chronic pancreatic, liver, or kidney disease; previous diagnosis of pancreatic cancer; history of pancreatic surgery; severe immune system disorders; and pregnancy in women.

### Definition of disease

WC was measured midway between the lower rib margin and the iliac crest in a standing position. Hypertriglyceridemia was defined as serum TG level ≥ 1.7 mmol/L. The participants were divided into four groups according to the WC and serum TG level^14^: (1) normal WC and normal TG (group-1; WC < 90 cm in men and < 80 cm in women, TG level < 1.7 mmol/L), (2) normal WC-elevated TG (group-2; TG level ≥ 1.7 mmol/L, WC < 80 cm in women and < 90 cm in men), (3) elevated WC-normal TG (group-3; WC ≥ 80 cm in women and ≥ 90 cm in men, TG level < 1.7 mmol/L), and (4) elevated WC-elevated TG (HTWP group, group-4; WC ≥ 90 cm in men and ≥ 80 cm in women, blood TG level ≥ 1.7 mmol/L). Additionally, the WC-TG index (WTI) was calculated as WC (cm) × TG level (mmol/L)^31^.

Body mass index (BMI) ≥ 28 kg/m^2^ was defined as obesity^32^. FL and FP were evaluated using trans-abdominal ultrasonography, as described previously^6^. A representative photograph of FP is shown in Supplementary figure S1. The diagnostic criteria for diabetes, hypertension, and MetS were based on previous studies^33,34^.

### Statistical analysis

IBM SPSS 20.0 version software (Inc., Chicago, IL) was used for statistical analysis. Continuous variables are expressed as mean ± standard deviation, and categorical variables are expressed as n (percentage) or median (interquartile range). The basic characteristics and differences among groups were compared using analysis of variance for continuous variables. Classification variables were analysed using the χ^2^ test. A p value of < 0.05 (two-sided) was considered statistically significant. To further investigate the relationship between HTWP and FP, we used WTI to predict FP using receiver operating characteristic (ROC) curve analysis. We calculated the best cut point according to the Youden index (= sensitivity − [1 − specificity]).

## Results

### Baseline clinical characteristics in different phenotype groups

A total of 1241 participants were included in the current study. The mean age of the participants was 45.7 ± 14.1 years, and 581 (46.8%) were men. The participants were divided into four groups according to WC and TG level: group-1 (n = 538), group-2 (n = 134), group-3 (n = 290), and group-4 (HTWP group, n = 279). A total of 307 (24.7%) participants were diagnosed with MetS. The prevalence of FP was 4.3% respectively (Table 1).

**Table 1:** Baseline clinical characteristics by the 4 phenotype groups.

	Total	G-1	G-2	G-3	G-4	P^a^
N	1241	538	134	290	279	
Male	581/46.8	220/40.9	97/72.4*	104/35.9*^‡^	160/57.3*&	< 0.001
Age	45.7 ± 14.1	41.0 ± 13.5	46.1 ± 12.1*	48.9 ± 14.0*	51.2 ± 13.6*^‡^	< 0.001
BMI	23.9 ± 3.5	21.6 ± 2.3	23.2 ± 2.2*	26.0 ± 3.0*^‡^	26.8 ± 2.9*^‡&^	< 0.001
WC	83.5 ± 10.7	75.2 ± 6.7	81.8 ± 6.4*	90.2 ± 7.9*^‡^	93.6 ± 7.3*^‡^^&^	< 0.001
Smoking	247/19.9	85/15.8	38/28.4*	46/15.9^‡^	78/28.0*^‡&^	< 0.001
Drinking	247/19.9	72/13.4	45/33.6*	44/15.2^‡^	86/30.8*^‡&^	< 0.001
MetS	307/24.7	0/0	28/20.9*	44/15.2*	235/84.2*^‡^^&^	< 0.001
HBP	98/7.9	13/2.4	8/6.0*	33/11.4*	44/15.8*^&^	< 0.001
T2DM	10/0.8	3/0.6	0/0	3/1.0	4/1.4	0.381
FL	366/29.5	43/8.0	44/32.8*	99/34.1*	180/64.5*^‡&^	< 0.001
FP	53/4.3	3/0.6	2/1.5	19/6.6*^‡^	29/10.4*^‡&^	< 0.001

Values are expressed as means (standard deviation) or n/%

G-1 (group-1): normal waist-normal triglyceride; G-2 (group-2): normal waist-elevated triglycerides; G-3 (group-3): elevated waist-normal triglycerides; G-4 (group-4): elevated waist-elevated triglyceride (HTGW).

*BMI* body mass index, *HBP* high blood pressure, *MetS* metabolic syndrome, *T2DM* type 2 diabetes mellitus, *FP* fatty pancreas, *FL* fatty live, *WC* waist circumference.

^a^Comparison among three groups.

*Compared with G-1, P < 0.05.

^‡^Compared with G-2, P < 0.05.

^&^Compared with G-3, P < 0.05.

The baseline characteristics of the participants are shown in Tables 1 and 2. Participants with HTWP (group-4) had a higher mean age (51.2 vs 41.0, 46.1, and 48.9 years), BMI (26.8 vs 21.6, 23.2, and 26.0 kg/m^2^), and WC (93.6 vs 75.2, 81.8, and 90.2 cm) than the other three phenotype groups, and group-4 also had a higher proportion of men than group-1 and group-3 (57.3% vs 40.9% and 35.9%) (all *p* < 0.001, Table 1). Moreover, individuals with HTWP had high levels of indicators related to metabolic disorder, including aspartate aminotransferase, alanine aminotransferase, gamma glutamyl transferase, serum uric acid, fasting blood glucose, TG, cholesterol, and low-density lipoprotein (all *p* < 0.0001, Table 2). Conversely, lower levels of high-density lipoprotein were observed in the HTWP group (*p* < 0.0001, Table 2). In addition, the proportions of participants with smoking and alcohol drinking habits were significantly higher in group-4 than in the other groups (all *p* < 0.001, Table 1).

**Table 2:** Laboratory data by the 4 phenotype groups.

	Total	G-1	G-2	G-3	G-4	P^a^
TG	1.6 ± 1.5	1.0 ± 0.3	2.6 ± 1.2*	1.2 ± 0.3*^‡^	3.1 ± 2.2*^‡^^&^	< 0.001
TC	4.4 ± 0.9	4.1 ± 0.7	4.6 ± 1.1*	4.5 ± 0.8*	4.7 ± 0.9*^&^	< 0.001
LDL-C	2.5 ± 0.7	2.3 ± 0.6	2.6 ± 0.9*	2.6 ± 0.7*	2.6 ± 0.7*	< 0.001
HDL-C^#^	1.2 ± 0.3	1.4 ± 0.3	1.1 ± 0.3*	1.3 ± 0.3^‡^	1.0 ± 0.3*^&^	< 0.001
FPG	5.2 ± 1.2	4.9 ± 0.6	5.4 ± 1.5*	5.3 ± 1.0*	5.7 ± 1.7*^&^	< 0.001
ALT	25.0 ± 19.0	18.9 ± 13.0	29.8 ± 17.5*	26.6 ± 18.6*	32.8 ± 25.0*^&^	< 0.001
AST	21.2 ± 10.1	18.8 ± 7.5	22.3 ± 7.3*	21.5 ± 9.5*	25.2 ± 14.1*^‡^^&^	< 0.001
γ-GGT	30.0 ± 33.1	18.4 ± 15.0*	43.8 ± 47.9*	28.8 ± 26.9*^‡^	46.5 ± 44.4*^&^	< 0.001
SCR	74.3 ± 33.3	74.0 ± 42.3	77.1 ± 18.4	71.5 ± 31.2	76.2 ± 15.3	0.265
SUA	308.4 ± 87.5	282.0 ± 79.1	333.9 ± 84.2*	303.1 ± 83.4*^‡^	352.5 ± 88.4*^&^	< 0.001

Values are expressed as means (standard deviation).

G-1 (group-1): normal waist-normal triglyceride; G-2 (group-2): normal waist-elevated triglycerides; G-3 (group-3): elevated waist-normal triglycerides; G-4 (group-4): elevated waist- elevated triglyceride (HTGW).

*TG* triglyceride, *TC* total cholesterol, *LDL-C* low density lipoprotein-cholesterol, *HDL-C* high density lipoprotein-cholesterol, *FPG* fasting plasma glucose, *ALT* aspartate aminotransferase, *AST* alanine aminotransferase, *γ-GGT* γ-glutamyltransferase, *SCR* serum creatinine, *SUA* serum uric acid.

^a^Comparison among four groups.

*Compared with G-1, P < 0.05.

^‡^Compared with G-2, P < 0.05.

^&^Compared with G-3, P < 0.05.

^#^Because of information incomplete, total number = 1228.

### Prevalence of FP and metabolic related diseases in the four subgroups

As shown in Table 1, group-4 had the highest prevalence of MetS (84.2%), which showed a significant difference from group-1, group-2, and group-3 (0%, 20.9%, and 15.2%, all *p* < 0.001). Similarly, the prevalence of FP (10.4%), FL (64.5%), and hypertension (15.8%) in the HTWP group were higher than those in the other three phenotype groups. The prevalence of diabetes in the four groups gradually increased with more phenotypic abnormality; however, no statistical significance was found.

### Association of the HTWP with FP

**Figure 1:**
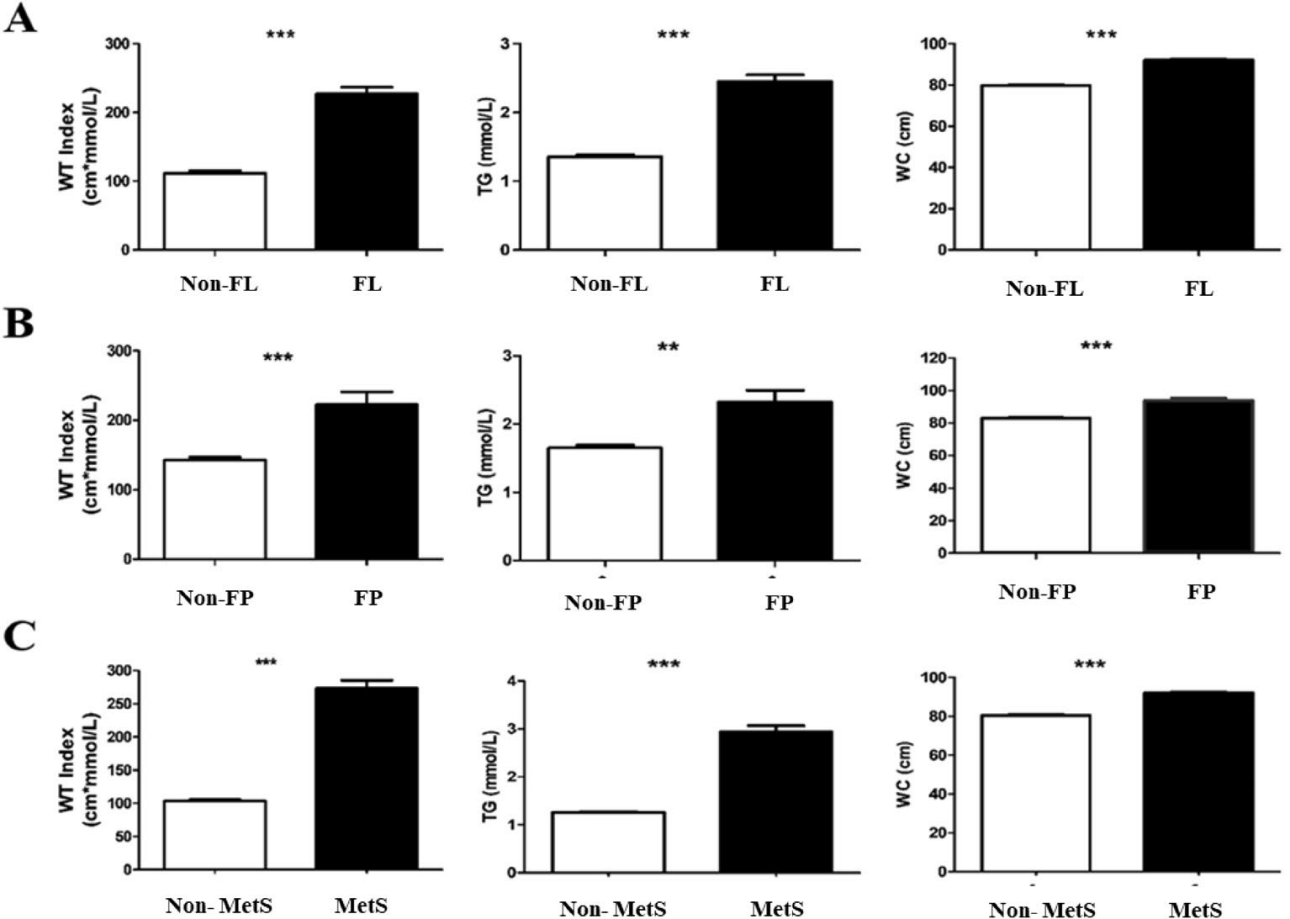
The histogram of WC, TG and WT index level between individuals with or without FL, FP and MetS. *WC* waist circumference, *TG* triglyceride, *WT index* waist circumference-triglyceride index, *FP* fatty pancreas, *Non-FP* non-fatty pancreas, *FL* fatty liver, *Non-FL* non-fatty liver, *Mets* metabolic syndrome. **p < 0.01, ***p < 0.001.

To further analyse the correlation between FP and HTWP, we divided the study population into different groups (Table 3, Fig. 1A). We quantified HTWP using the WTI. The occurrence rate of HTWP and the WTI were significantly higher in the FP group than in the non-FP group (54.7% vs 21.0%, 222 ± 135 vs 142 ± 141, both *p* < 0.001). In addition, we found that the prevalence of the elevated WC-normal TG phenotype (group-3) was higher (p < 0.05) than that of the healthy phenotype (group-1). Moreover, no significant difference was observed between group-1 and group-2 (normal WC-elevated TG group). Furthermore, we assessed the relationship of two other metabolic diseases (FL and MetS) to WTI (Fig. 1B,C). The FL group also showed a higher proportion of participants with HTWP, higher prevalence of MetS, and higher WTI than the non-FL group (*p* < 0.001).

**Table 3:** Comparison of phenotype distribution and WTI between different diseases.

	Non-FP	FP	P	Non-FL	FL	P	Non-Mets	Mets	P
	n = 1188	n = 53		n = 875	n = 366		n = 934	n = 307	
G-1	535/45.1	3/5.7	< 0.001	495/56.6	43/11.7	< 0.001	538/57.6	0/0	< 0.001
G-2	132/11.1	2/3.8		90/10.3	44/12.0		106/11.3	28/9.1	
G-3	271/22.8	19/35.8		191/21.8	99/27.1		246/26.4	44/14.3	
G-4 (HTGW)	250/21.0	29/54.7		99/11.3	180/49.2		44/4.7	235/76.6	
WT index	142 ± 141	222 ± 135	< 0.001	112 ± 104	227 ± 179	< 0.001	104 ± 67	274 ± 212	< 0.001

Values are expressed as n/% or means (standard deviation).

G-1 (group-1): normal waist-normal triglyceride; G-2 (group-2): normal waist-elevated triglycerides; G-3 (group-3): elevated waist-normal triglycerides; G-4 (group-4): elevated waist- elevated triglyceride (HTGW).

WT index, waist circumference (cm) × triglyceride index (mmol/L).

*HBP* high blood pressure, *FP* fatty pancreas, *FL* fatty live, *Mets* metabolic syndrome.

### ROC curve: WT index predicts the FP

**Figure 2:**
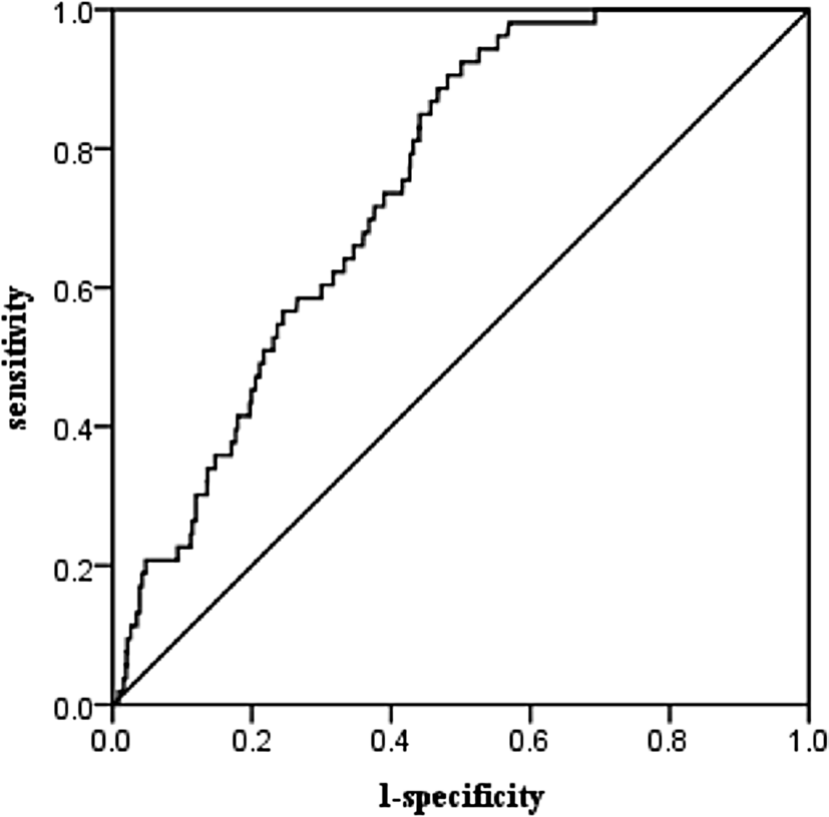
ROC analysis for the WT Index and FP. *ROC* receiver operator characteristic, *WT Index* waist circumference-triglyceride index.

In the ROC curve of the WTI as a predictor of FP, the area under the curve was the largest (0.746) with a WTI of 107.09, with a corresponding sensitivity and specificity of 90.6% and 51.9%, respectively (Fig. 2).

## Discussion

To date, no specific biomarkers for FP have been identified. Studies have demonstrated that trans-oesophageal ultrasonography, CT, and MRI are accurate methods for diagnosing FP^35–37^. Saisho et al.^38^ calculated the fat/parenchyma ratio using CT, and the results were not significantly different from histological and anatomical findings, clearly suggesting the sensitivity of CT in the diagnosis of FP. In addition, CT and MRI have good consistency. Previous studies have reported that the location of fat infiltration is consistent in patients with FP diagnosed using CT and MRI. Currently, CT images are still commonly used to evaluate the presence of FP in the pancreas and spleen in research studies. Although CT and MRI are better methods for detecting FP, ultrasonography is the preferred large-scale screening method in daily clinical practice owing to its lower financial and time costs^39^. Jeong et al. demonstrated that FP can be diagnosed using trans-abdominal ultrasonography in 16.5% of cases, similar to the diagnosis rates reported in other cohort studies in the United States^39^, reflecting the feasibility of trans-abdominal ultrasonography.

FP is a common clinical phenomenon that is closely related to obesity, diabetes, and MetS. With increasing depth of research, clinicians’ understanding of FP has gradually increased in recent years; however, there is still a large gap compared with the knowledge of FL. Additionally, studies have shown that FP is closely related to pancreatitis (acute and chronic) and pancreatic cancer, suggesting that FP may contribute to the progression of a series of pancreatic diseases. Therefore, earlier detection of FP is an urgent clinical issue.

The current study shows a clear correlation between visceral fat, waist circumference and FP^39,40,43^. We have previously demonstrated that only central obesity, but not general obesity, is an independent risk factor for FP^6^. The results suggest that a simple, easily accessible measurement of visceral obesity may have a better predictive ability for FP. HTWP, a clinical indicator of visceral fat accumulation, was first proposed by Lemieux et al. in 2000. They showed that simultaneous measurement of WC and fasting TG could be used as an inexpensive screening method to identify men at a high risk for the atherogenic metabolic triad and cardiovascular diseases^14^. Since then, more studies have confirmed that HTWP is closely related to the occurrence of cardiovascular diseases^19,20^. With further supporting research, HTWP has been considered an inexpensive and effective marker reflecting visceral (intra-abdominal) obesity and metabolic disorders. It is particularly suitable for Asians who are generally underweight but prone to the development of visceral obesity.

The correlation between HTWP and FP has not been reported. In our study, the prevalence of FP was significantly higher in the HTWP group than in the three other phenotype groups, and the proportion of participants with HTWP was relatively higher in the FP group than in the non-FP group. We also found that the prevalence of the elevated WC-normal TG phenotype (group-3) was higher (*p* < 0.05) than that of the healthy phenotype (group-1). Moreover, no significant difference was observed between group-1 and group-2 (normal WC-elevated TG group). The co-existence of elevated WC and elevated TG level had a significant additive effect on the incidence of FP. This phenomenon may be closely related to the production of free fatty acids and a pancreatic lipotoxic effect in individuals with obesity; however, the specific mechanism needs to be further clarified. In 2015, Yang et al.^31^ combined WC and TG into WTI for the first time, showing that WTI is a better indicator of the cardiovascular event risk in patients with coronary heart disease. Whether HTWP can be used as a risk factor for predicting FP has not been reported. In this study, we used WTI as a quantitative indicator to further predict FP. ROC curve analysis showed that the maximum area under the curve was 0.746, and the results were statistically significant. The above results all indicated a clear correlation between HTWP and FP.

Limited clinical epidemiology studies show that the incidence rate of FP is 10–35%^9,11–13,41,42^. According to a new study by Ural Koç et al.^43^, FP was determined visually in 29.8% of hospitalized patients (30.6% in men and 29% in women) by non-contrast CT, in addition, the visual assessment graded of FP steatosis was almost none to mild. The incidence rate of FP in this study was 4.3%, which is lower than that reported in previous studies. Clinical studies have shown that metabolic diseases and old age are independent risk factors for FP. However, in the present study, the average age of the population was 45.71 ± 14.16 years. The incidence rates of underlying diseases such as diabetes and hypertension were significantly lower than those in previous large epidemiological studies in China^44^. This may be because our study population comprised hospital staff with relatively good health. All of the above-mentioned causes eventually led to a lower incidence rate of FP in our group.

Our study had several limitations. First, all participants were health examiners from a city hospital, which resulted in a limited scope of sample collection. Second, as mentioned above, FP was diagnosed using trans-abdominal ultrasonography, which is not a perfect diagnostic method for this condition and may underestimate some of FP in none to mild visual assessment grade. Nevertheless, trans-abdominal ultrasonography is suitable for the health screening of large sample populations and has the advantage of being a non-invasive, fast, and economical diagnostic tool. In practice, the diagnosis and definition of FP may vary among different sonographers, which may cause bias in the data. Therefore, our ultrasound diagnosis and definition seem to be crucial. Third, our method was based on a cross-sectional study, and the causal relationship between HTWP and FP has not been determined. Fourth, the study population was obtained from a physical examination population. It was difficult to obtain accurate serological indicators of diabetes, such as serum insulin and C-peptide levels, especially in some individuals with prediabetes. Therefore, data related to the development of diabetes and decreased insulin secretion are lacking. To our knowledge, this is the first study to provide novel evidence of an association between HTWP and FP. More clinical studies are needed to verify our results.

## Conclusion

HTWP is closely associated with FP, and WTI could be used as a preliminary screening tool for FP.

## Supplementary Information

Supplementary Information
