# Supplementary material for: Relationship between fatty pancreas and hypertriglyceridemic waist phenotype: a cross-sectional study
Source: Sci Rep. 2020 Dec 14;10:21937. doi: 10.1038/s41598-020-78883-1 (PMC7736283; doi:10.1038/s41598-020-78883-1)
Supplement: Supplementary file 1 — Supplementary Information [file 41598_2020_78883_MOESM1_ESM.pdf]

Relationship between Fatty Pancreas and Hypertriglyceridemic Waist Phenotype: A Cross-sectional Study

Xiaoping Yu [1,2,3*], Dan Wang[1,4*], Weiming Xiao[1,4*], Xinlin Shi[1], Qiang She [1,4], Hongguang Sun[3], Tingyue Qi[3], Renyan Xu[2,3], Guiqing Li [1,4], Xinnong Liu[1], Weijuan Gong [1,4,5], Zhigang Yan[1,4#], Yanbing Ding [1,4#], and Guotao Lu [1,4#]

1. Institute of digestive diseases, Affiliated Hospital of Yangzhou University, Yangzhou University, Jiangsu, China.
2. Department of Health Promotion Center, Affiliated Hospital of Yangzhou University, Yangzhou University, Jiangsu, China.
3. Department of Ultrasound, Affiliated Hospital of Yangzhou University, Yangzhou University, Jiangsu, China.
4. Pancreatic Center, Department of Gastroenterology, Affiliated Hospital of Yangzhou University, Yangzhou University, Jiangsu, China.
5. Jiangsu Co-innovation Center for Prevention and Control of Important Animal Infectious Diseases and Zoonoses, College of Veterinary Medicine, Yangzhou, 225001, People's Republic of China.

*** Xiao-ping Yu, Dan Wang and Wei-ming Xiao contributed equally to this work.**

Correspondence to: Zhi-gang Yan: jsyzyzg@aliyun.com; Yan-bing Ding: ybding@yzu.edu.cn; Guo-tao Lu: gtlu@yzu.edu.cn; No. 386 Hanjiang Median Road, Yangzhou 225000, Jiangsu, China.

Telephone: +86-0514-82981199 to 81950.

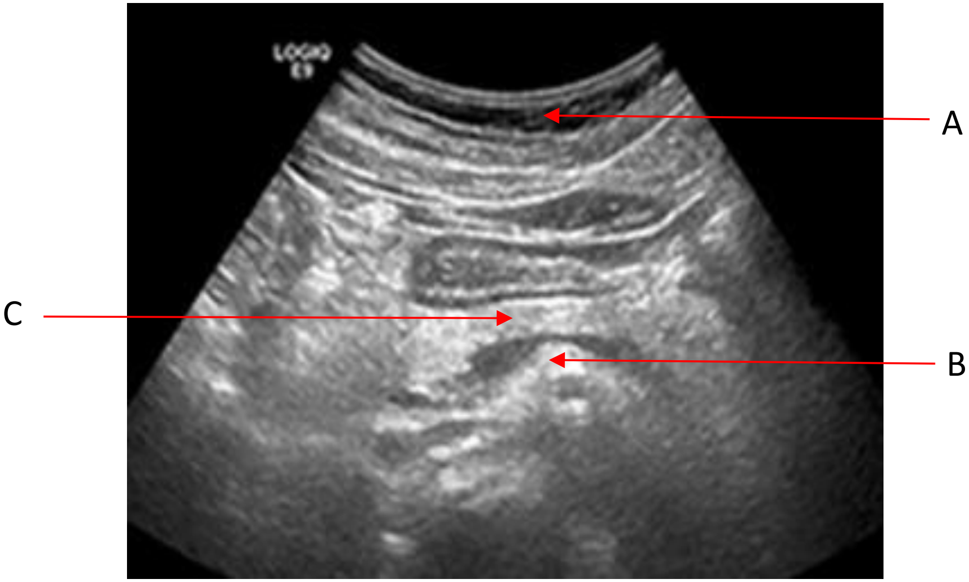

Fig S1.1 Non- fatty pancreas with normal body mass index and waist circumference.

A. Subcutaneous fatty tissue.

B. Adipose tissue in the area of the superior mesenteric artery.

C. Pancreas.

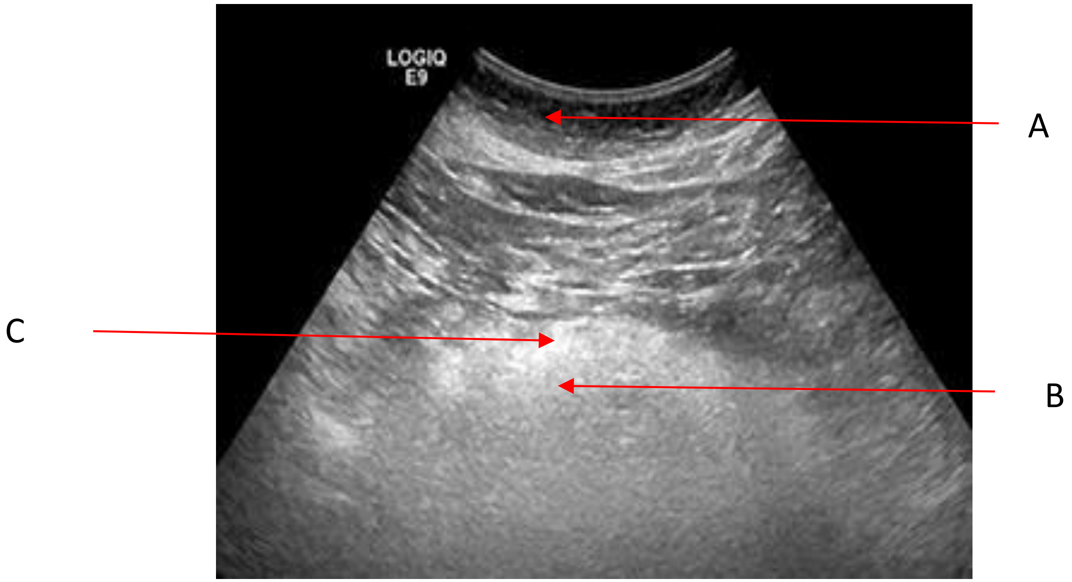

Fig S1.2 Fatty pancreas with normal body mass index and waist circumference.

A. Subcutaneous fatty tissue.

B. Adipose tissue in the area of the superior mesenteric artery.

C. Pancreas.

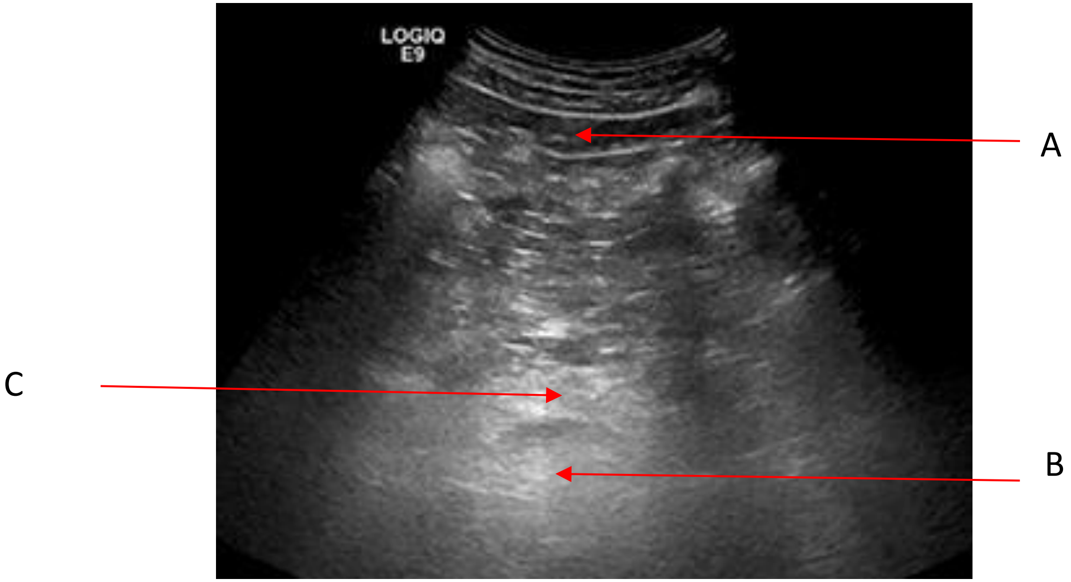

Fig S1.3. Non-fatty pancreas with central obesity.

A. Subcutaneous fatty tissue.
B. Adipose tissue in the area of the superior mesenteric artery.
C. Pancreas.

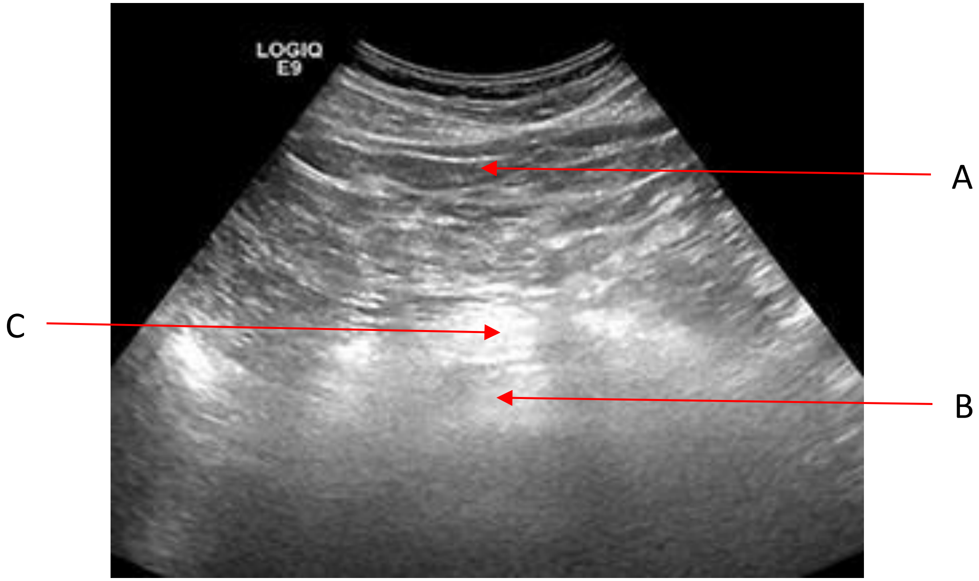

Fig S1.4. Fatty pancreas with central obesity.

A. Subcutaneous fatty tissue.
B. Adipose tissue in the area of the superior mesenteric artery.
C. Pancreas.
